# A novel encrypted traffic detection model based on detachable convolutional GCN-LSTM

**DOI:** 10.1038/s41598-025-13397-2

**Published:** 2025-07-29

**Authors:** Xiaogang Yuan, Jianxin Wan, Dezhi An, Huan Pei

**Affiliations:** School of Cyber Security, Gansu University of Political Science and Law, Lanzhou, Gansu China

**Keywords:** Malicious encrypted traffic, Intrusion detection, Detachable convolution, Graph neural network, Attention mechanism, Computer science, Information technology

## Abstract

With the widespread adoption of network encryption technologies, traditional detection methods increasingly struggle to identify malicious encrypted traffic due to their limited ability to capture structural and behavioral characteristics. To address this issue, this paper proposes a Detachable Convolutional GCN-LSTM (DC-GL) model. The proposed model constructs graph-structured data by integrating protocol-layer features and traffic statistical features extracted from encrypted flows. A Graph Convolutional Network (GCN) is employed to capture structural dependencies among nodes, while a Long Short-Term Memory (LSTM) network models the temporal dynamics of traffic behavior. To improve computational efficiency and feature extraction performance, detachable convolution is introduced into the GCN layers. In addition, an attention mechanism is incorporated to enhance the representation of critical features. Experimental results demonstrate that the DC-GL model outperforms several mainstream approaches in terms of accuracy, recall, and other key metrics, while also exhibiting faster convergence and greater robustness. These results suggest that DC-GL offers an effective and promising approach for malicious encrypted traffic detection.

## Introduction

In recent years, as more devices are connected to the internet, the volume of network data has grown significantly^1^. At the same time, with growing demand for data privacy protection, more services and applications have adopted encryption protocols for communication. This trend exacerbates network security challenges^2^. According to the Google Transparency Report, encrypted traffic accounted for 95% of the total data generated by all Google products and services as of January 2022. While encryption technologies offer strong protection for users’ data privacy, they have also been increasingly exploited by attackers to conceal communications between malicious programs and command-and-control servers, thereby evading firewalls and intrusion detection systems^3^. Traditional security detection methods are often inadequate for encrypted traffic. This poses significant threats to network security systems. With the widespread adoption of edge devices and fog computing, there is an increasing demand for higher detection efficiency to achieve real-time network protection^4^. Therefore, effectively identifying malicious encrypted traffic in high-load encrypted environments has become an urgent challenge^5^.

Current research on encrypted traffic classification mainly focuses on three approaches: Deep Packet Inspection (DPI), machine learning, and deep learning. DPI was an early technique that detected encrypted traffic by decrypting ciphertext segments within packets^6^. Lin et al. proposed a fingerprint-based method for detecting encrypted malicious traffic^7^. It uses Transport Layer Security (TLS) state transition sequences and packet statistical features to build attribute rules. The method combines a shortest-path graph kernel with locality-sensitive hashing (LSH) for efficient classification. Sun Zhongjun et al. introduced an algorithm based on DPI to inspect payload randomness^8^. They used DPI technology to quickly screen network traffic and applied information entropy calculation with Monte Carlo simulation to estimate classification errors. DPI methods typically rely on protocol-specific rule matching. In the early days, when encrypted traffic was relatively low, these methods could effectively analyze plaintext data to detect potential threats. However, with the growing variety of encryption protocols and frequent updates to encryption suites, rule-based matching approaches of traditional DPI methods have been significantly impacted and are no longer sufficient to cope with the complexity and evolution of modern encrypted traffic environments^9^.

To overcome the limitations of DPI, researchers have developed machine learning-based detection methods. Unlike DPI, these methods do not require extracting plaintext data. Instead, they rely on statistical features, byte distribution features, or TLS characteristics. Shen et al. proposed an unsupervised machine learning (ML) based malicious traffic detection system called HyperVision, which constructed a graph structure based on interaction patterns among network flows and identified abnormal behaviors using ML techniques^10^. The method does not rely on labeled attack data and achieves effective detection of unknown attacks through graph feature analysis, while balancing detection accuracy and efficiency. Zou Futai et al. proposed a detection algorithm using the Hidden Markov Model (HMM)^11^. This method identified malicious traffic by analyzing hidden state relationships. The performance of ML-based models relies heavily on the robustness of the employed handcrafted features, limiting their stability^12^. Attackers can evade detection by modifying their communication behavior or disrupting feature distributions. Furthermore, these methods struggle to capture structural information and temporal dependencies within traffic, limiting their accuracy and generalization ability.

Unlike traditional machine learning, deep learning can automatically learn effective features from raw data without relying on manual feature design or selection^13^. Cai et al. proposed a malicious traffic detection model called GSA-DT, which combined graph self-attention and decision trees^14^. The method builds a traffic graph to capture node relationships and uses a self-attention mechanism in the GCN. LeakyReLU is applied to mitigate gradient vanishing and neuron death. Wang Qinfan et al. developed a graph neural network (GNN) using a hierarchical graph pooling architecture^15^. This method constructed traffic interaction graphs and applied GCN to extract node features and topological information. However, this approach only considers structural information and neglects temporal features, resulting in incomplete feature extraction. Wang et al. proposed MTC, a multi-task model that combined Transformer and 1D-CNN for encrypted traffic classification^16^. The model adopted a parallel structure and used a feature fusion block to integrate local and global features. It performs application identification and traffic characterization simultaneously. Liu et al. proposed the TransECA-Net model, which integrated the ECA-Net module with convolutional neural networks to achieve efficient feature extraction and dimensionality reduction through a channel selection mechanism^17^. The model incorporated residual connections in the Transformer to ensure stable gradient flow. Yang Zhongfu et al. combined Capsule Networks (CapsNet) with Bidirectional Long Short-Term Memory Networks (Bi-LSTM) for encrypted traffic detection. CapsNet captured positional features, while Bi-LSTM extracted temporal features^18^. Although this method excels at recognizing traffic patterns, its overall complexity and dynamic routing mechanism reduce training efficiency and generalization capability. Li et al. proposed an encrypted traffic classification method based on Interaction Behavior Graphs (IBGC), which introduces interaction features to better distinguish similar traffic^19^. Packets are mapped to actions and modeled as attributed graphs. A subgraph sampling model was used to capture correlations between actions. The method performs well on public datasets. Additionally, current deep learning approaches typically emphasize either structural features or temporal dependencies, and rarely fully integrate both aspects simultaneously^20^. This limitation results in incomplete feature representation and restricts overall detection performance in encrypted traffic scenarios.

To address the challenges in encrypted traffic detection, this study proposes a DC-GL model, with a focus on enhancing both computational efficiency and feature representation. At the core of this architecture is the detachable convolution mechanism, which decomposes traditional convolution operations into depthwise and pointwise components^21^. This design significantly reduces computational complexity while enabling more effective extraction of localized features, making it particularly suitable for complex, high-dimensional traffic data. Building on this foundation, the model constructs graph-structured representations of encrypted traffic by integrating protocol-level fields and flow-level statistical features. The GCN component captures spatial dependencies by aggregating information from neighboring nodes, modeling protocol interactions and overcoming the limitations of traditional machine learning approaches that rely on handcrafted features. To complement structural modeling, the LSTM module captures temporal dependencies within encrypted flows, identifying evolving patterns and addressing the shortcomings of conventional GCNs, which often lack sequence modeling capabilities. Further enhancing the model, an attention mechanism is introduced to dynamically weigh node features according to their relevance, improving overall representation quality and detection performance.

The main contributions of this study are as follows:

- A DC-GL prediction model is constructed by integrating GCN and LSTM modules to jointly capture structural features and temporal dependencies in encrypted traffic, enhancing the representation of spatiotemporal patterns.
- The standard convolution in GCN is replaced with depthwise and pointwise convolutions, improving local feature extraction efficiency, reducing model complexity, and accelerating convergence during training.
- An attention mechanism is introduced to dynamically assign weights based on the importance of node features, leading to more discriminative node embeddings and improved detection performance.

The rest of this paper is organized as follows. Section “Encrypted traffic detection model” designs the Encrypted Traffic Detection Model based on Detachable Convolutional GCN-LSTM. Section “Experimental results and analysis” is the experimental part, which introduces the experimental design and specific content and analyzes the experimental results in detail. Finally, section Conclusion” presents conclusions and future research directions.

## Encrypted traffic detection model

**Fig. 1:**
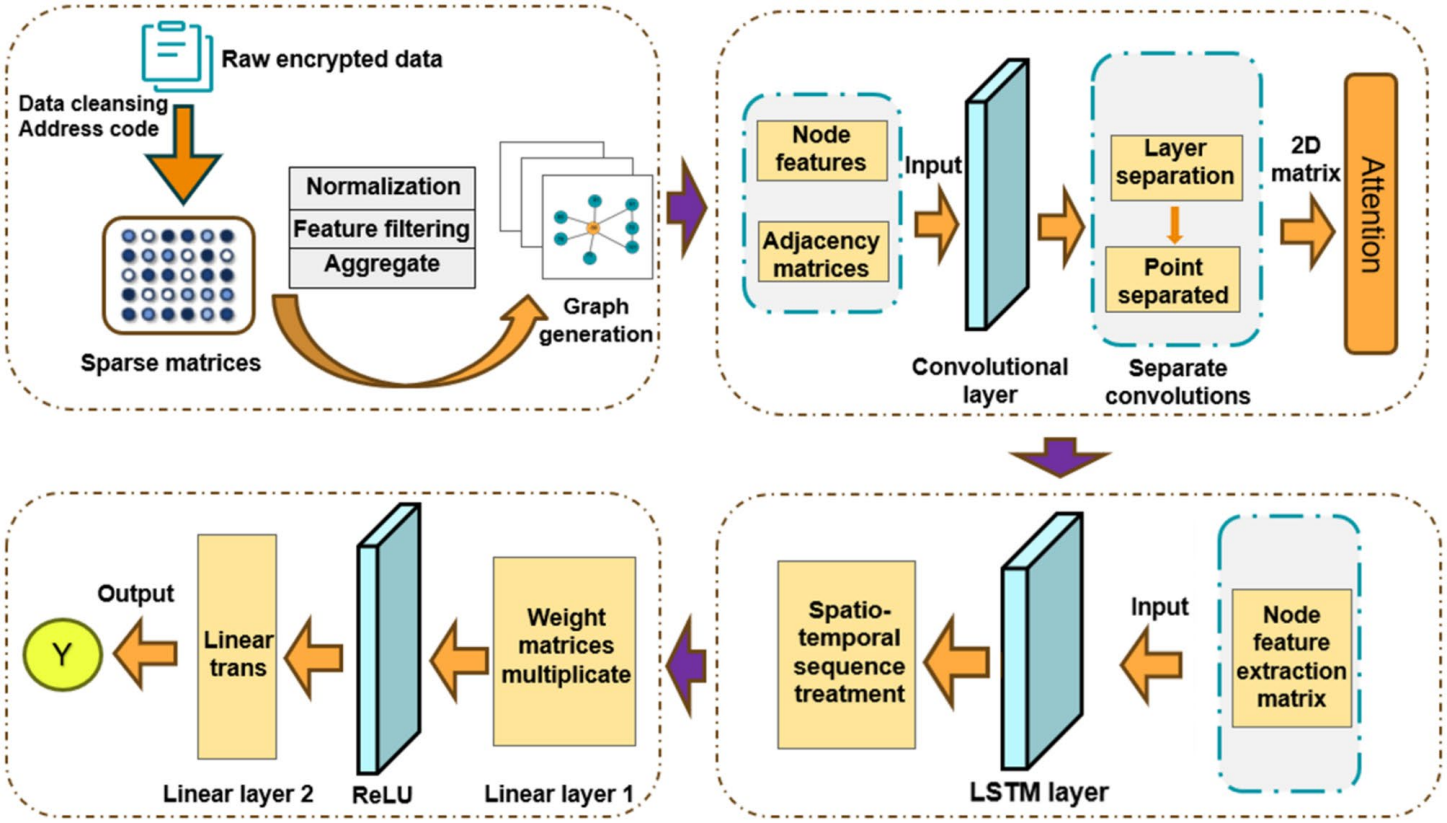
Architecture of the proposed DC-GL model.

This paper presents the DC-GL model, which constructs graph-structured data from encrypted traffic flows. During preprocessing, non-encrypted traffic is removed, and both statistical and protocol-level features are extracted. Nodes are assigned weighted feature vectors, and edge weights reflect interaction strength between flows. Detachable convolution is applied in the GCN to improve local feature extraction, while the LSTM captures temporal patterns. A multi-layer perceptron (MLP) is used for final classification. The overall architecture is illustrated in Fig. 1.

### Data preprocessing and feature engineering

Network traffic is a collection of data packets aggregated according to the five-tuple information (source address, destination address, source port number, destination port number, and transmission protocol) and arranged in chronological order^22^. This set includes not only the data to be transmitted but also partial control information. Each intercepted packet contains these five critical pieces of information^23^.

After aggregation based on the five-tuple and chronological order, preprocessing is performed, including the removal of outliers and filtering to retain only SSL/TLS-related encrypted flows^24^. The data is then converted into a structured file format to facilitate further analysis. For records with incomplete or missing information, zero-padding is applied to fill missing values, ensuring that data gaps do not negatively impact model training. Numerical features are normalized using min-max scaling to map their values into the [0,1] range, which unifies feature scales and prevents training biases caused by differing magnitudes. Categorical features are encoded using appropriate methods such as one-hot encoding or label encoding, enabling the model to effectively interpret and utilize these discrete variables.

Malicious communication behaviors display distinctive patterns that can be detected within network traffic^25^. Therefore, this study focuses on analyzing the transmission patterns of encrypted traffic to identify potential malicious activities^26^. These characteristics can be broadly categorized into two types: flow statistical features and encryption-related protocol features. The overall feature system is summarized in Table 1.

**Table 1:** Overview of extracted feature categories.

Feature Type	Feature Name	Feature Type	Feature Name
Traffic Statistics	Packet Length	TLS Features	Version
	Session Interval		Cipher
	Flags		SNI
	Port Activity		ALPN
	Byte Ratio		Cert Chain Info
	···		···

For behavioral features, the extracted indicators include packet length distribution, session interval time, and the activity frequency of source and destination IP addresses and ports. Compared to Benign encrypted communication, malicious flows often exhibit fixed packet length patterns, more frequent interactions, or abnormal address access patterns. These statistical features provide critical clues for identifying anomalous behaviors.

At the protocol level, the extracted features include the TLS version, cipher suite, Server Name Indication (SNI), certificate fields, JA3 fingerprint, Application-Layer Protocol Negotiation (ALPN) fields, and certificate chain structure. These are essential components of the encryption handshake process and effectively represent the identities, encryption strategies, and application contexts of the communicating hosts.

### Graph structure generation

After preprocessing, a proximity matrix is constructed using source and destination IP indices. However, relying solely on a single type of feature (such as IP addresses or port numbers) may introduce bias into the graph structure and limit its ability to represent complex relationships. To mitigate this issue, our method integrates protocol fields with statistical behavioral features during graph construction to build a multi-level semantic representation. This effectively weakens the dominance of any individual feature and more comprehensively captures high-order relationships among nodes, thereby enhancing the robustness and expressiveness of the graph structure.

**Fig. 2:**
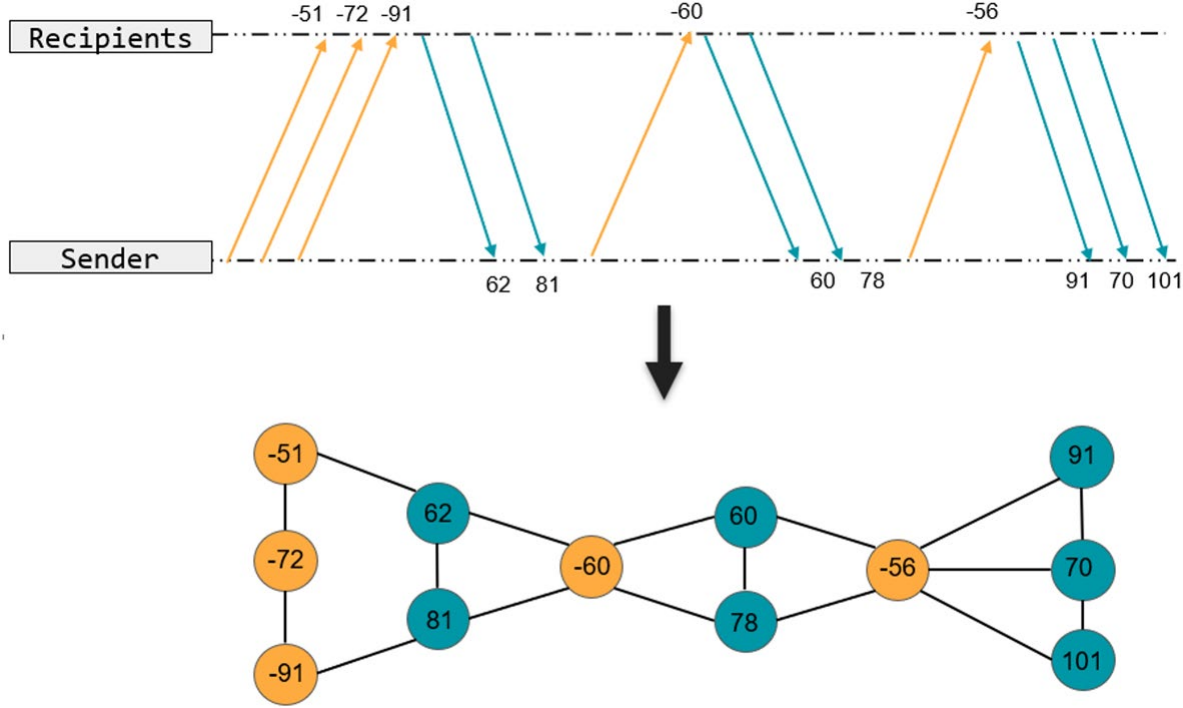
Interaction graph construction graph.

To represent the encrypted traffic as a graph, each node corresponds to a single encrypted traffic packet. These nodes carry multi-dimensional feature vectors encompassing protocol attributes (e.g., protocol version, SNI), statistical properties (e.g., packet size, inter-arrival time), and identity characteristics (e.g., counts of source and destination IPs). For each processed batch, nodes are indexed by unique source-destination IP pairs to reflect communication entities within the batch. The number of nodes, n, in each graph corresponds to the unique source-destination IP pairs within the batch, usually ranging from several hundred to over a thousand, depending on the data distribution. Accordingly, the adjacency matrix is initialized as a sparse *n*×*n* matrix representing the connections among these nodes. This batching strategy balances graph granularity and computational efficiency. The topological relationships between nodes are illustrated in Fig. 2.

The graph scale is determined by the batch size, with batches typically consisting of 1000 encrypted traffic samples. For each processed batch, nodes are indexed by unique source-destination IP pairs to represent the communication entities within the batch. The number of nodes n in each graph corresponds to the unique source-destination IP pairs within the batch, usually ranging from several hundred to over a thousand, depending on data distribution. Accordingly, the adjacency matrix is initialized as a sparse n×n matrix representing the connections among these nodes. This batching strategy balances graph granularity and computational efficiency.

Subsequently, a sparse adjacency matrix is created to represent node connections and is then converted to a dense form. Row sums are incremented by 1 and transformed using an inverse square root scaling for normalization. The computation formulas are as follows.

1 $${S_i}={(1+\sum\limits_{{j=1}}^{n} {{a_{ij}}} )^{ - 0.5}}$$

Where *S*_i_ representing the *i*-th element of the array, *i* represents the row index of the transformed dense matrix *A*. The *j* represents the column index of the transformed dense matrix *A*. *a*_ij_ represent the connection between nodes *i* and *j*.

Then, the last normalization step of the adjacency $D^{{ - \tfrac{1}{2}}}$ is obtained, and the normalized *A* is denoted${A_{norm}}$. After the expansion ${A_{norm}}$, the calculation formula for all the elements within the array is shown as follows.

2 $${A_{norm}}(i,j)=\sum\limits_{{k=1}}^{n} {\sum\limits_{{l=1}}^{n} {{D_{ik}}^{{ - \frac{1}{2}}}} } {a_{kl}}{D_{lj}}^{{ - \frac{1}{2}}}$$

After normalization, the adjacency matrix no longer allows high-degree nodes to dominate information flow, enabling more balanced contributions from nodes of varying degrees^27^. This strategy stabilizes training and can be further improved with graph regularization techniques that constrain node relationships during learning^28^. Each node in the graph can be represented as a feature vector in a vector space, where *V* denotes the set of nodes and E represents the associated attributes. Encrypted traffic features are integrated into these nodes, and each node $\vec {A}(\nu )$ is encoded as:

3 $$\vec{A}(\nu ) = \left[ {\begin{array}{*{20}c} {f_{1} (\nu ),f_{2} (\nu ), \ldots ,f_{n} (\nu )} \\ \end{array} } \right]$$

Where, ${f_1}(\nu )$ represents a given attribute value. When ${f_1}(\nu )$ representing the source IP number feature.

Each node represents a packet with encrypted traffic features, where each dimension of the feature vector corresponds to a specific attribute (such as port activity)^29^. By analyzing attributes like source addresses, the distribution of communicating devices can be observed. To emphasize important features under different scenarios, a weight factor is assigned to each dimension. The weighted feature vector of node ${\vec {A}_{\omega ,\gamma }}(\nu )$ is computed as:

4 $${\vec {A}_{\omega ,\gamma }}(\nu )=\frac{1}{\gamma }[{\omega _1}{f_1}(\nu ),{\omega _2}{f_2}(\nu ), \cdots ,{\omega _n}{f_n}(\nu )]$$

Where, ${\omega _n}$ is the weight factor corresponding to the *n*-th encrypted traffic feature. ${f_n}(\nu )$ is the *n*-th encrypted traffic characteristic value corresponding to the node. $\gamma$ represents the normalization factors.

Edges in the graph represent associations between nodes *u* and *v* capturing topological relationships within the network. The interaction strength of each edge is quantified based on selected traffic features and their corresponding weights. The calculation formula is as follows.

5 $$w(e)=k \cdot \frac{{{g_1}{{(e)}^\alpha } \cdot \prod\limits_{{i=3}}^{m} {{g_i}} {{\left( e \right)}^{{\gamma _i}}}}}{{{g_2}{{(e)}^\beta }}}$$

Here, *k* is a scaling constant set to 1 to normalize the edge weight magnitude. The parameters α, β, and $\:{\gamma\:}_{i}$ are fixed in this work as α = 1, β = 1, and $\:{\gamma\:}_{i}$=0.5, aiming to balance the contribution of connection-related features, penalize dominant high-magnitude terms, and regulate auxiliary traffic attributes.

The graph construction method shares similarities with classical techniques such as Multidimensional Scaling (MDS) and spectral clustering, as all utilize similarity or affinity matrices to model pairwise relationships. However, there are notable differences: the graph construction process does not solely rely on Euclidean distances or spectral eigenvectors, but incorporates semantically defined edge weights specific to encrypted traffic. This enhances the expressiveness of the graph representation.

### Attention mechanism

**Fig. 3:**
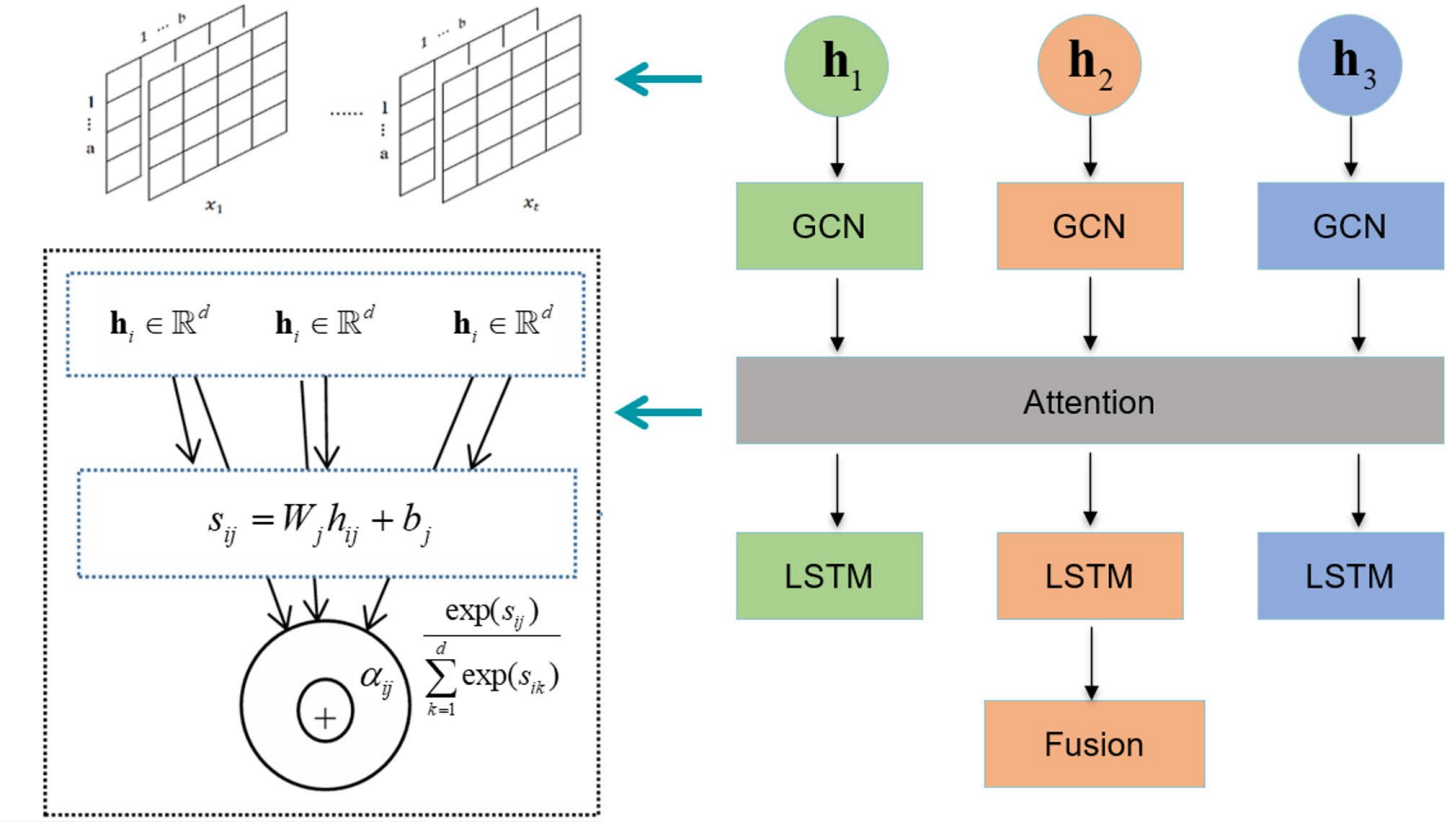
Attention mechanism.

GCN updates node features by aggregating information from neighboring nodes. However, different feature dimensions may contribute unequally to the node representation. To address this, a node-level attention mechanism is introduced to adaptively weigh each feature dimension of every node. This allows the model to dynamically adjust attention based on the input data and graph structure. The overall attention workflow is illustrated in Fig. 3.

First, a learnable weight matrix and bias vector are applied to the GCN output node feature vectors via a linear transformation, producing preliminary attention scores for each feature dimension. This operation can be expressed as:

6 $${S_{i,j}}={W_j} \cdot {x_{i,j}}+{b_j}$$

where ${S_{i,j}}$ is the attention score for node *i* at feature dimension *j*, and *W*_*j*_, *b*_*j*_ are learnable parameters.

Attention scores are computed through a learnable weight matrix and bias, then normalized via the softmax function so that each node’s attention weights sum to 1. The corresponding calculation is shown below.

7 $${\alpha _{ij}}=\frac{{\exp ({s_{ij}})}}{{\sum\limits_{{k=1}}^{d} {\exp } ({s_{ik}})}}$$

Where, exp() is a natural exponential function. ${S_{ij}}$is the element of column *j*, row *i*, in the attention score matrix *S*.

The normalized weights ${\alpha _{ij}}$are applied element-wise to the node features to recalibrate them, amplifying important features and suppressing less relevant ones. This enables GCN to selectively emphasize key information during feature aggregation.

### Detachable convolution

To improve the efficiency and representational capacity of the model when processing high-dimensional encrypted traffic features, detachable convolution is integrated into the GCN. The standard graph convolution operation is decomposed into two steps: first, a depthwise convolution is independently applied to each input channel, followed by a pointwise convolution (1 × 1 kernel) that fuses information across all channels.

In the implementation, the detachable convolution uses a kernel size of 3 × 1, corresponding to a local window across the temporal or feature dimension to effectively capture local sequential patterns. The ratio between depthwise and pointwise channels is approximately 1:1, meaning each input channel has a dedicated depthwise convolution kernel, while the pointwise convolution integrates features from all channels.

**Fig. 4:**
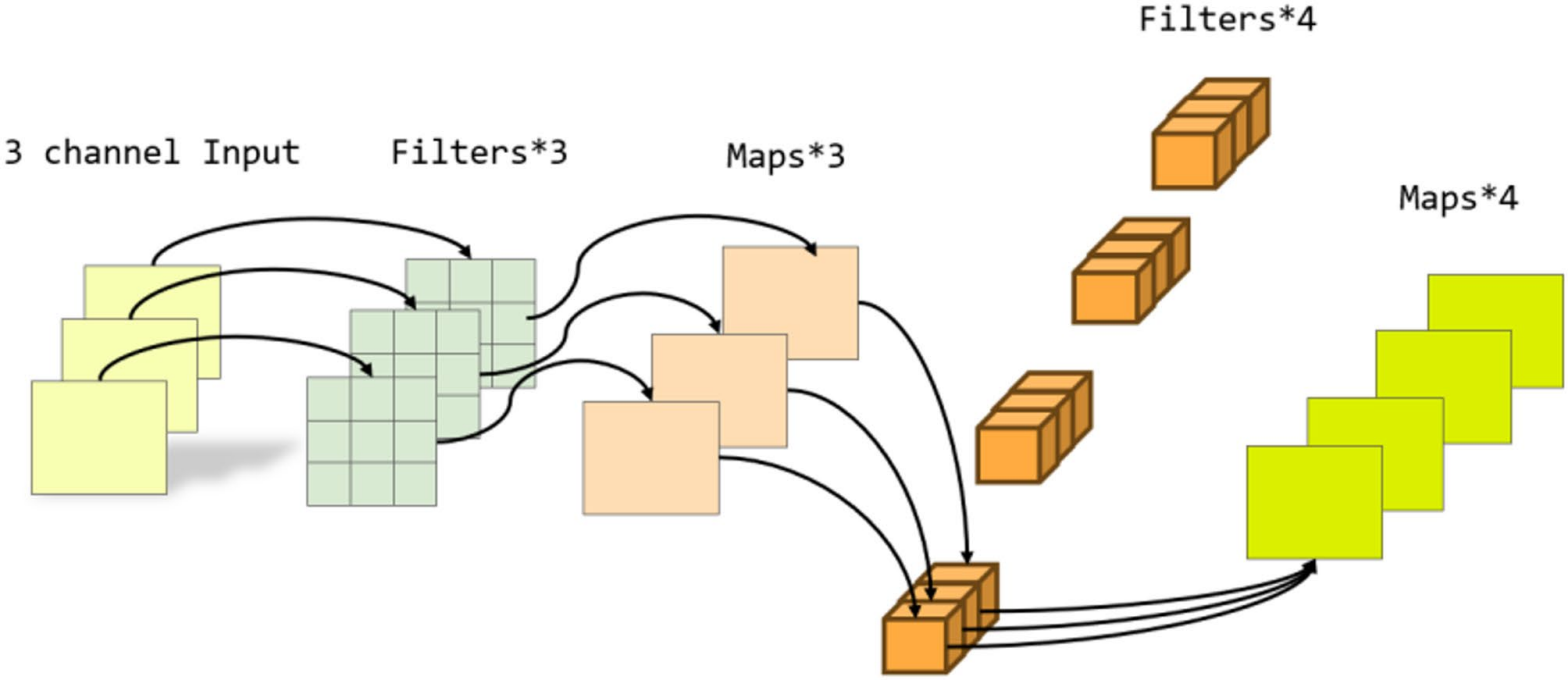
Detachable convolutional graph layer structure.

This configuration maintains detection accuracy while substantially reducing the number of parameters and computational complexity, leading to improved training efficiency. The structure of the detachable convolution layer is illustrated in Fig. 4.

When processing encrypted traffic, the detachable convolution is first applied to node features. It performs convolution independently on each input channel using a predefined kernel. The input consists of a batch of encrypted traffic samples, where each sample had multiple feature channels and a time-series length. At a specific position, the output for channel c is calculated as follows.

8 $${Y_{c,i,j}}=\sum\limits_{{m=0}}^{{\ker nelsize - 1}} {\sum\limits_{{n=0}}^{{\ker nelsize - 1}} {{X_{c,i+m - padding,j+n - padding}}} } \times {K_{d,m,0,c}}$$

Here, ${X_{c,i+m - padding,j+n - padding}}$ denotes the value of the *c*-th channel in the input data, and ${K_{d,m,0,c}}$ is the corresponding depthwise convolution kernel. Indices *m* and *n* refer to positions within the kernel, and padding is applied to handle edge cases.

After depthwise convolution, pointwise convolution is applied to fuse information across all channels at each position, enabling interaction between different encrypted traffic features. The final output ${Z_{i,j}}$ at a certain position ${(i.j)_{}}$ is computed as shown in the following formula.

9 $${Z_{i,j}}=\sum\limits_{{c=0}}^{{channels - 1}} {{Y_{d,i,j,c}}} \times {K_{p,0,c,0}}$$

**Fig. 5:**
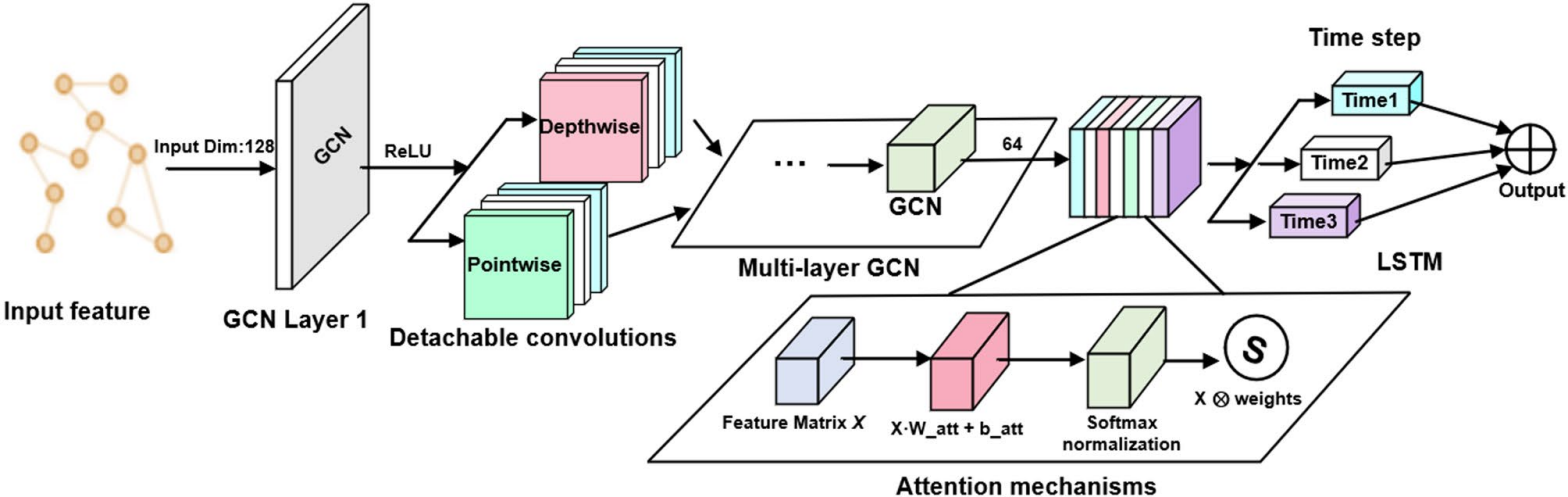
Overall architecture.

Through pointwise convolution, the features extracted by detachable convolution across different channels are fused and recombined. The final output thus contains both local features from individual channels and integrated features across channels. The overall architecture of the proposed model is illustrated in Fig. 5.

**Fig. 6:**
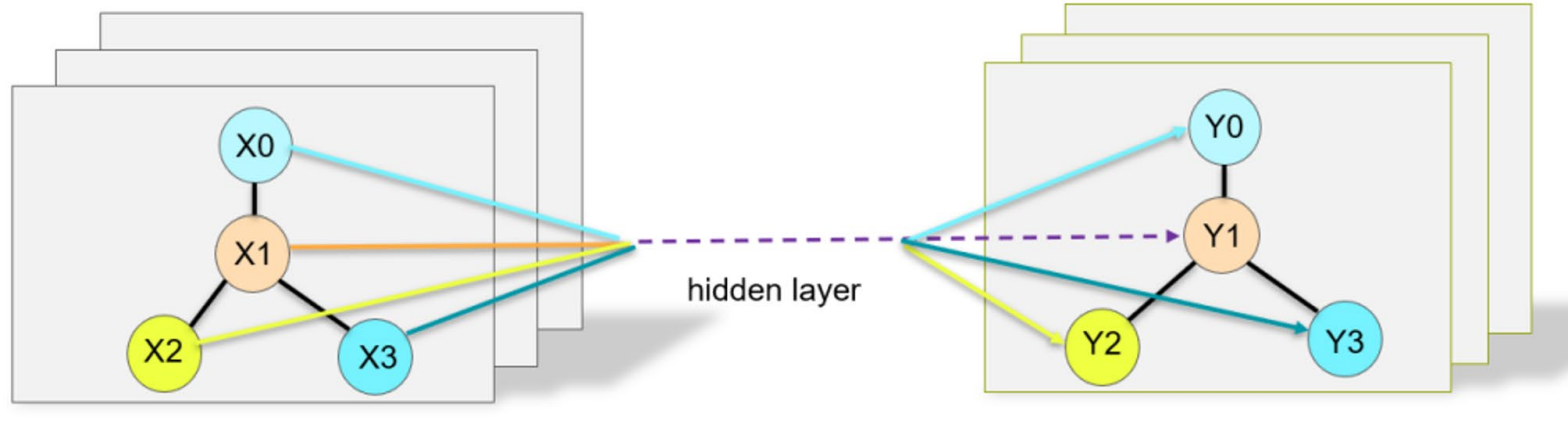
Topology diagram.

This enhances the model’s ability to learn generalizable representations, improving its adaptability and accuracy when handling encrypted traffic from diverse sources and formats. In addition, during the convolution process from X to Y across GCN layers, the connectivity between nodes remains unchanged, as illustrated in Fig. 6.

### Output of the classification results

In graph convolutional processing, node features often evolve over time or in sequence. The LSTM layer effectively captures such temporal dependencies by remembering long-term patterns. In network traffic analysis, node features may form time series as traffic evolves. By modeling these changes, the LSTM layer helps the model better understand dynamic network behaviors.

LSTM enhances Recurrent Neural Network (RNN). It performs better compared to other neural networks because of its property of remembering patterns selectively for a long period of time^30^. The LSTM introduces the concept of gating and constructs three gates, namely the input gate *i*_*t*_, the forget gate *f*_*t*_, and the output gate *O*_*t*_, as well as an internal memory cell *C*_*t*_^31^.

In the LSTM layer, the forgetting gate *f* determines which less important flow feature information needs to be forgotten. The calculation formula is shown as follows.

10 $${f_\iota }=\sigma ({W_f} \times [{h_{\iota - 1}},{x_\iota }]+{b_f})$$

Where, $\sigma$is the sigmoid function, which compresses the output values between 0 and 1. ${W_f}$is the weight matrix of the forgetting gate. ${h_{\iota - 1}}$ was the hidden state of the last moment. $x_{t}$ is the input to the current moment. ${b_f}$ is the bias term of the forgetting gate.

The input gate determines which new information will be added to the cell state, with the calculation formula shown as follows.

11 $${i_t}=\sigma \left( {{W_i} \times \left[ {{h_{t - 1}},{x_t}} \right]+{b_i}} \right)$$

Combine the output information of the previous moment and the current input data. The state vector *C*_*t*_ of the LSTM cell will be updated accordingly. This enables the cell state to store long-term memory information and maintain the network’s dynamic memory ability. The update formula is shown as follows.

12 $${C_t}={f_t} \times {C_{t - 1}}+{i_t} \times \tanh \left( {{W_c} \times \left[ {\begin{array}{*{20}{c}} {{h_{t - 1}},{x_t}} \end{array}} \right]+{b_c}} \right)$$

The output gate *O*_*t*_ determines which information in the cell state will be output, with the formula shown as follows.

13 $${O_t}=\sigma ({W_o} \times [{h_{t - 1}},{x_t}]+{b_o})$$

The LSTM layer processes sequences from the GCN, integrating structural and temporal features into richer representations. Given the complexity of encrypted traffic, a simple linear classifier is insufficient. Therefore, MLP is used to further process the LSTM output X, which captures key structural and sequential patterns. Through a layer of linear mapping and non-linear activation, hidden feature relationships are highlighted, and after a second linear transformation, the output Y is generated for final classification.

## Experimental results and analysis

### Experimental environment and parameter setting

#### Experimental environment

The experiments were conducted using Python 3.8 with PyTorch as the neural network framework^32^, implemented in the PyCharm IDE. The details of the computer hardware platform are listed in Table 2.

**Table 2:** Experimental platform configuration.

Name	Parameter
CPU	Intel E5-2670
GPU	NVIDIA RTX 4060
RAM	64 GB
Python	3.8
CUDA	11.2

#### Data set

Data set plays a significant role in the evaluation of an efficient anomaly detection technique^33^. This study used the CICIDS-2017 and CIC-AndMal2017 datasets^34,35^. CICIDS-2017 contains seven days of network traffic with various attacks (e.g., DoS, brute force, port scan), and includes TLS/SSL-encrypted traffic, making it suitable for this task. CIC-AndMal2017 includes both Benign and malicious apps from Google Play and malware sources, covering encrypted and unencrypted traffic. Dataset statistics are summarized in Table 3.

**Table 3:** Dataset Configuration.

CICIDS-2017			AndMal2017		
Label	Name	Number	Label	Name	Number
Attack	FTP-Patator	3212	BENIGN	BENIGN	7589
	PortScan	2907	Scareware	Scareware	3291
	DoS Golden	12,311	SMS Malware	SMS Malware	2734
	Brute Force	4081	Adware	Adware	2150
	SSH-Patator	2678	Ransomware	Ransomware	1076
Normal	BENIGN	103,089			

For both CICIDS-2017 and CIC-AndMal2017 datasets, the data were split into training and testing sets with a ratio of 70–30%. The split was performed randomly while fixing the random seed at 42 to ensure reproducibility and consistent evaluation across experiments. This approach preserves the overall data distribution while enabling reliable performance comparison.

#### Experimental parameters

To ensure consistent and fair evaluation across different model architectures and datasets, all experiments were conducted under a unified set of training configurations. The selection of hyperparameters was guided by prior work on graph-based and sequence-based learning, as well as by empirical tuning to achieve stable convergence across diverse traffic patterns.

The hyperparameters cover key components such as graph convolutional layers, LSTM units, and classification heads, as well as training controls including learning rate, batch size, and regularization. A summary is provided in Table 4.

**Table 4:** Parameter setting table.

Name	Description	Default Value
feats_per_node	Dimensionality of initial node input features	200
num_gcn_layers	Number of graph convolutional layers	4
lstm_feats	Hidden state size of the LSTM module	32
num_lstm_layers	Number of LSTM layers	1
cls_in_feats	Input dimensionality of the final classification layer	64
batch_size	Number of graphs processed per training batch	16
learning_rate	Initial learning rate used for optimization	0.001
dropout_rate	Dropout rate applied to neural layers	0.3
weight_decay	L2 regularization coefficient to prevent overfitting	5e-4

All models were trained using the Adam optimizer with parameters set as β₁ = 0.9, β₂ = 0.999, and ε = 1e-8. For the smaller AndMal2017 dataset, the number of training epochs was increased appropriately to ensure sufficient model fitting, while for the larger CICIDS-2017 dataset, fewer epochs were used to reduce computational cost and mitigate overfitting risk.

#### Evaluation indicators

In network traffic detection, model performance was typically evaluated using accuracy, recall, F1-score, ROC curve, and Area Under the Curve (AUC). Accuracy measures overall correctness, recall reflects the ability to detect intrusions, and F1-score balances precision and recall. The ROC curve shows the trade-off between true and false positive rates, while AUC summarizes overall classification performance.

14 $$Acc=\frac{{TP+TN}}{{TP+TN+FP+FN}}$$

15 $$Recall=\frac{{TP}}{{TP+FN}}$$

16 $$F1=2 \cdot \frac{{Precisio{n_{macro}} \cdot Recal{l_{macro}}}}{{Precisio{n_{macro}}+Recal{l_{macro}}}}$$

Where True Positive (TP) is the number of correctly predicted positive samples, True Negative (TN) is the correctly predicted negatives, False Positive (FP) is the number of false positives, and False Negative (FN) is the number of false negatives.

### Comparison of detachable convolution effectiveness

#### Comparison of detachable convolution effectiveness

**Fig. 7:**
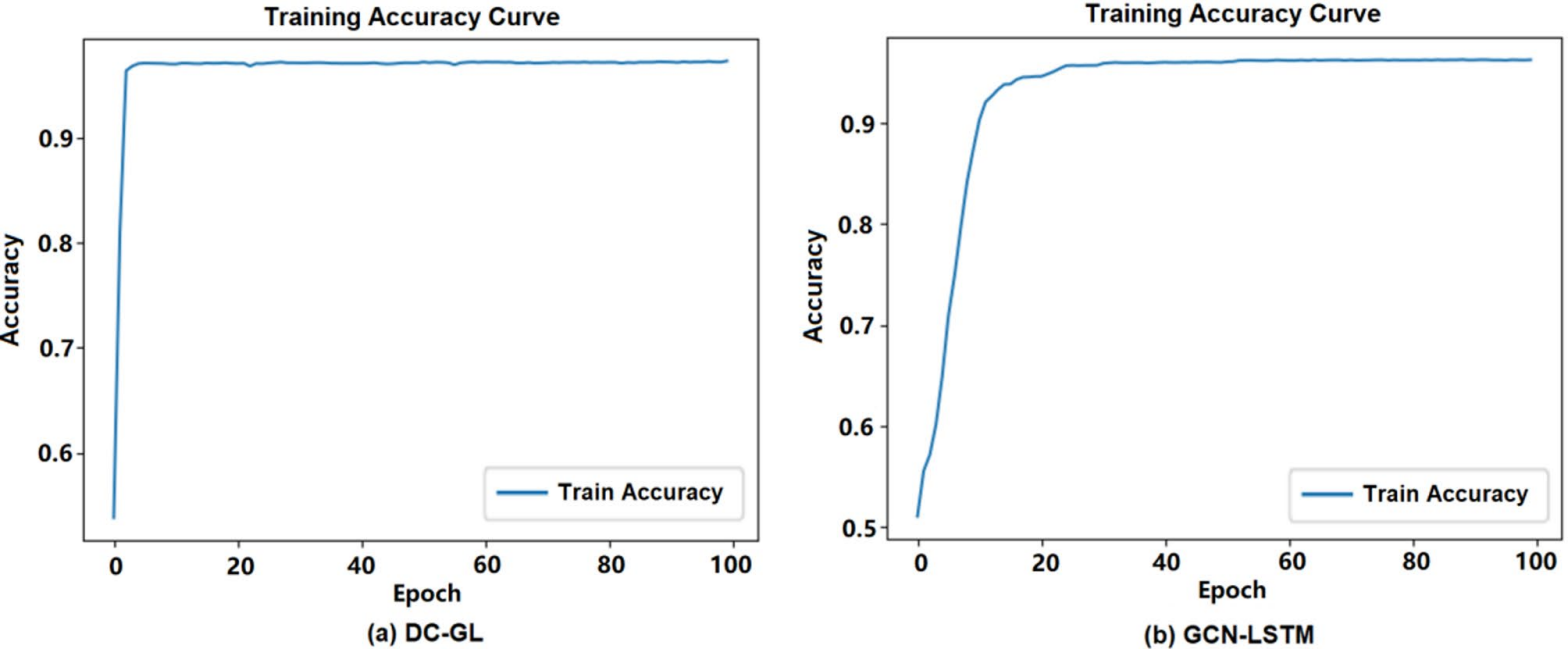
Experimental results of the accuracy curve.

**Fig. 8:**
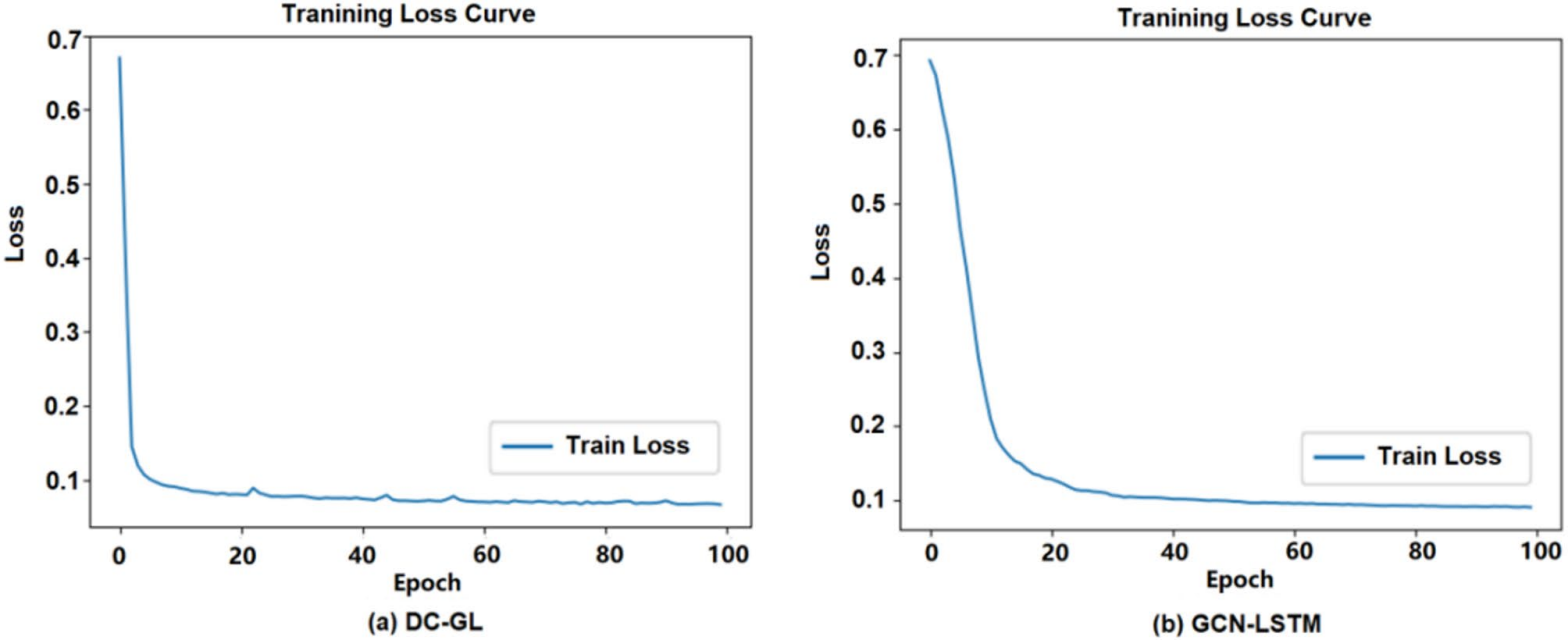
Experimental results of the training loss curve.

Traditional convolution processes all channels together, while detachable convolution divides this into depthwise and pointwise steps. To evaluate its effectiveness, we compared the traditional GCN-LSTM model with the DC-GL model under the same hardware and identical Adam optimizer settings, ensuring a fair comparison. Experimental results are shown in Figs. 7 and 8, where (a) refers to the DC-GL model and (b) refers to the GCN-LSTM model.

Figure 7 displays the accuracy curves during training. According to Fig. 7a, the DC-GL model reached an accuracy of about 97% by around 20 epochs and then remained relatively stable. Figure 7b shows that the standard GCN-LSTM model required about 30 epochs to achieve a comparable level of accuracy. This observation implies that DC-GL could effectively extract key features at an earlier stage, leading to a faster convergence rate. Figure 8 illustrated the training loss curves. As shown in Fig. 8a, the DC-GL model’s loss decreased rapidly within the first 20 epochs and stabilized at approximately 0.1. In contrast, Fig. 8b indicates that the standard GCN-LSTM model needed around 35 epochs to reach a similarly stable loss value. The introduction of the detachable convolution mechanism in DC-GL enables more efficient extraction of local features required for this task, thereby improving training efficiency.

The experimental results showed that by embedding the detachable convolution operation into the GCN-LSTM model, the DC-GL model performed better than the traditional GCN-LSTM model on key indicators such as accuracy, convergence rate, and AUC, verifying the effectiveness of the detachable convolution technique.

#### Ablation experiments

To better understand the contribution of each core component in the DC-GL model, we conducted an ablation study to evaluate the impact of the attention mechanism, temporal modeling, and detachable convolution on the overall detection performance. Three variant models were constructed by removing one component at a time and compared against the complete DC-GL model. The results on the CICIDS-2017 and AndMal2017 datasets were summarized in Table 5.

**Table 5:** Table of results of ablation experiments.

Method	CICIDS-2017			AndMal2017		
	Acc	Recall	F1-score	Acc	Recall	F1-score
DC-GL	97.29%	97.01%	97.15%	97.82%	97.58%	97.67%
w/o Attention	96.58%	96.13%	96.35%	96.94%	96.62%	96.76%
w/o LSTM	95.37%	94.98%	95.17%	95.84%	95.41%	95.62%
w/o Detachable Conv.	96.92%	96.44%	96.68%	97.10%	96.85%	96.97%

The results indicated that removing the LSTM module led to the most noticeable performance degradation, particularly in recall, which reflected the importance of modeling sequential behavior in encrypted traffic analysis. Excluding the attention mechanism also results in moderate performance decline. This suggested that attention helps highlight salient features and enhances representation quality, but is somewhat complementary to the temporal and spatial modeling. On the other hand, removing the detachable convolution module led to only a slight reduction in performance. This indicated that while detachable convolution improves computational efficiency and local feature extraction, its effect on classification accuracy is less pronounced.

Overall, the ablation results confirm that all components contribute positively to model performance. However, the temporal modeling and attention mechanism were particularly essential for effective encrypted traffic detection, while detachable convolution primarily optimizes resource usage and model scalability.

#### Comparison of convolutional layer parameters

**Fig. 9:**
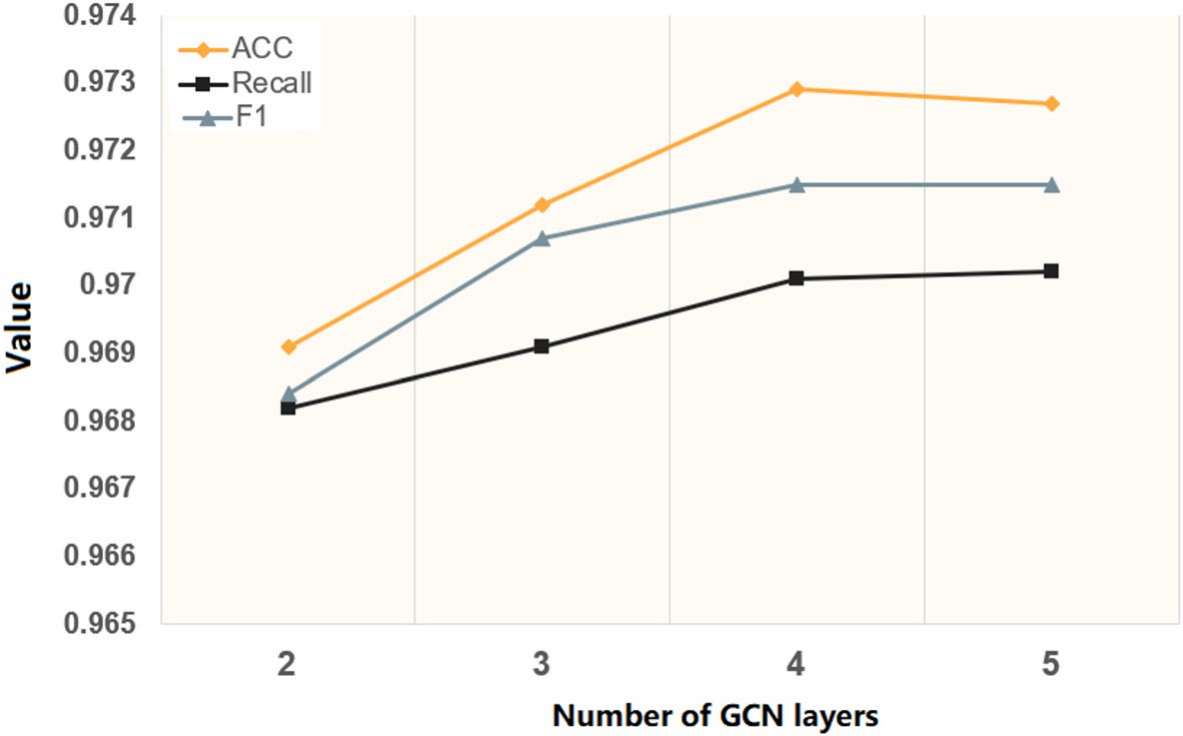
Results of the GCN experiments with different numbers of layers.

In the encrypted traffic detection experiment, we evaluated the impact of different numbers of GCN layers on model performance. The DC-GL model was trained with 2, 3, 4, and 5 GCN layers, while keeping other parameters unchanged to ensure fair comparison. The results are shown in Fig. 9.

As the number of GCN layers increased, the training accuracy improved initially and then stabilized. With 2 layers, the accuracy was 96.9%; it rose to 97.1% with 3 layers, and stabilized around 97.3% with 4 or more layers. This suggests that increasing the GCN depth helps the model to better learn encrypted traffic features up to a certain point, after which the benefit plateaus.

F1 scores followed a similar trend: 96.8% with 2 layers, 97.0% with 3 layers, and about 97.1% with 4 or more layers. These results have showed that deeper GCNs improve stability and feature extraction, as they better capture the spatial relationships between packets in network sessions. However, adding layers beyond 4 provides limited additional gains.

### DC-GL model detection performance analysis

#### Comparison of test indicators

To systematically evaluate the performance of the DC-GL model in encrypted traffic detection, nine representative baseline models covering three distinct modeling paradigms were selected.

Sequence-based models focus on capturing temporal dependencies in traffic data and include HMM, CNN-LSTM, and CapsNet-BiLSTM^18^. Graph structure-based models leverage the inherent relational information in network traffic and comprise GraphSAGE, GCN^15^, Contextual Temporal Graph Convolutional Network (CT-GCN), and DyGCN-LSTM^36^, which integrate dynamic graph convolution with LSTM to model evolving graph-structured sequences. Transformer-based models, such as Transformer-CNN^16^ and TransECA-Net^17^, employ self-attention mechanisms to capture long-range dependencies and complex feature interactions within the traffic data.

This comprehensive selection of baselines enabled a thorough assessment of the effectiveness and robustness of DC-GL across diverse modeling approaches. Results are presented in Table 6.

**Table 6:** Comparison results with other methods.

Method	CICIDS-2017			AndMal2017		
	Acc	Recall	F1-score	Acc	Recall	F1-score
DC-GL	**97.29%**	**97.01%**	**97.15%**	**97.82%**	**97.58%**	**97.67%**
GraphSAGE	94.52%	94.21%	94.26%	94.93%	94.67%	94.73%
CNN-LSTM	95.10%	94.88%	94.99%	95.48%	95.34%	95.41%
GCN	93.98%	93.28%	93.61%	94.34%	93.95%	94.11%
CapsNet-BiLSTM	96.85%	96.81%	96.78%	97.23%	97.09%	97.16%
CT-GCN	96.07%	95.80%	95.83%	96.47%	96.15%	96.31%
HMM	93.83%	93.63%	93.73%	94.11%	93.87%	93.98%
DyGCN-LSTM	96.85%	96.62%	96.70%	97.34%	97.12%	97.20%
Transformer-CNN	96.48%	96.21%	96.30%	96.90%	96.66%	96.72%
TransECA-Net	96.12%	95.98%	96.01%	96.70%	96.44%	96.52%

Bold values indicate the maximum value of the corresponding performance indicator.

On the CICIDS-2017 dataset, the DC-GL model achieved an accuracy, recall, and F1-score of 97.29%, 97.01%, and 97.15%, respectively. These results were higher than those of all comparison methods. Compared with GraphSAGE, the improvements in accuracy, recall, and F1-score were 2.77%, 2.80%, and 2.89%, respectively. This indicates that DC-GL had a stronger capability to aggregate neighborhood features and preserve multi-dimensional information.

Compared with CNN-LSTM, the DC-GL model performed better in modeling structural traffic features, thanks to its use of graph neural networks. CNN-LSTM, which lacked graph structure awareness, was limited in capturing spatial dependencies in encrypted traffic.

The GCN model did not incorporate temporal modeling and thus could not effectively extract time-dependent traffic features. By integrating the LSTM module, DC-GL captured temporal variations and improved its classification performance.

The CapsNet-BiLSTM model achieved detection results close to DC-GL. However, its complex architecture led to lower feature fusion efficiency. The use of detachable convolution in DC-GL improved feature extraction accuracy and overall model effectiveness.

CT-GCN showed stable results in general but performed less consistently when dealing with frequent updates in graph structures. Its convergence and detection stability were weaker than those of DC-GL. As a result, its accuracy, recall, and F1-score were lower.

The HMM model showed the weakest performance on both datasets. Due to its reliance on fixed probabilistic transitions, it could not effectively capture high-dimensional and nonlinear features in encrypted traffic. This highlights the limitations of traditional statistical models in handling modern encrypted traffic.

The DyGCN-LSTM model incorporated both graph structure and temporal dependencies and achieved competitive results, but it still lagged slightly behind DC-GL. This is partly because DC-GL used depthwise separable convolution, which could extract more fine-grained features.

The Transformer-CNN model combined self-attention with 1D convolution to capture both global and local dependencies. While it performed well overall, its lack of explicit graph modeling led to weaker spatial structure learning, resulting in slightly lower performance than DC-GL.

TransECA-Net integrated multi-scale temporal convolution with cross-attention mechanisms, achieving relatively high accuracy. Nevertheless, its performance remained slightly lower than DC-GL due to its weaker capacity for graph-guided spatial reasoning.

In summary, the DC-GL model achieved the best performance across all three metrics on both datasets. By combining graph convolution, temporal modeling, efficient detachable convolution, and attention-based adaptive weighting, it delivered strong and stable results in multi-class encrypted traffic detection.

#### Multi-class detection performance comparison

**Fig. 10:**
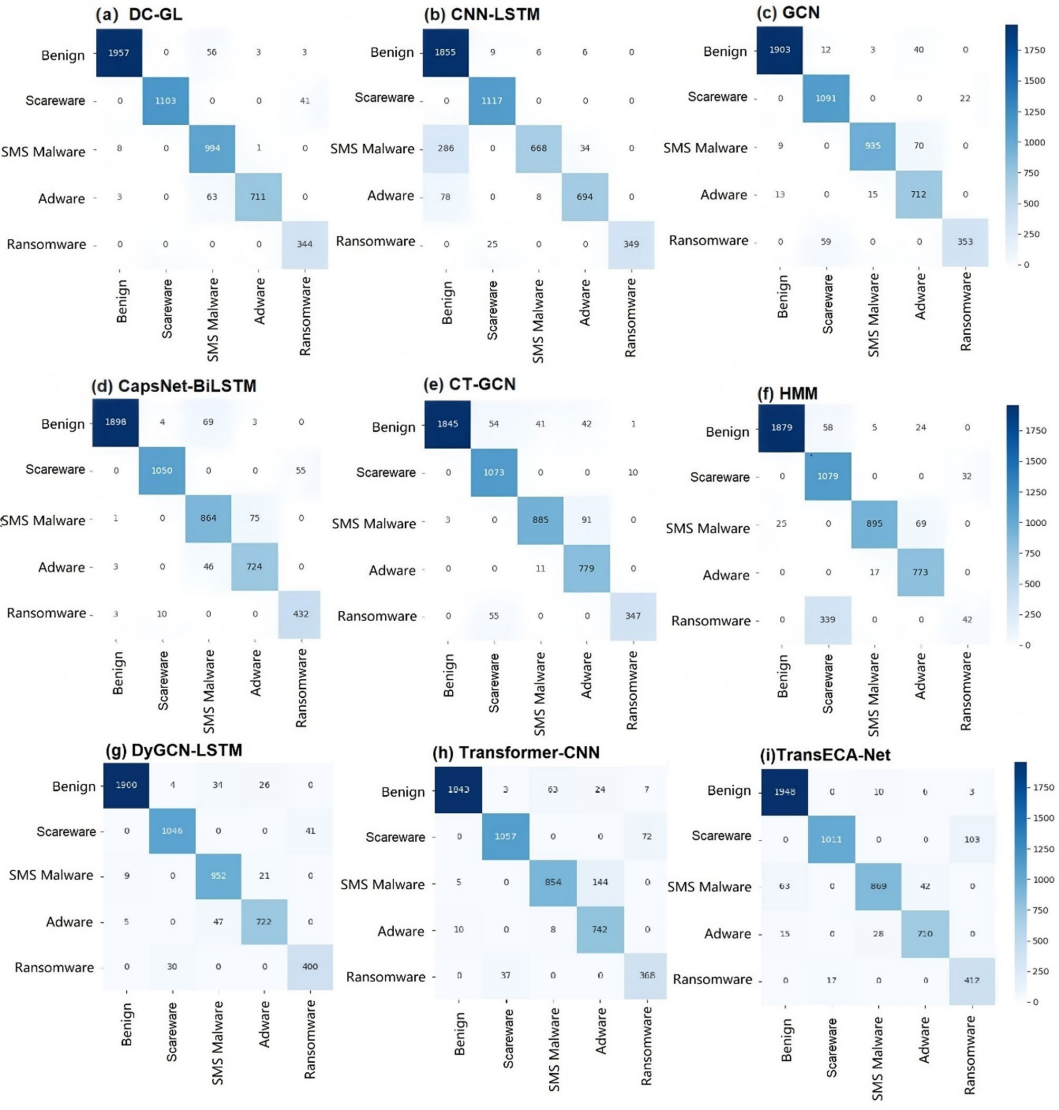
Confusion matrix of the experimental results.

To further evaluate the effectiveness of the proposed DC-GL model in multi-class encrypted traffic detection, comparative experiments were conducted against several representative baseline approaches. Confusion matrices based on the CIC-AndMal2017 dataset were used to visualize and compare the classification performance across different traffic categories, as shown in Fig. 10.

The DC-GL model exhibited consistently stable classification performance across most traffic categories. It accurately identified Benign, Scareware, Adware, and Ransomware classes while maintaining low misclassification rates in the SMS Malware category. The confusion between SMS Malware and other categories was minimal, indicating that DC-GL effectively captured subtle feature differences.

In contrast, the CNN-LSTM model confused the BENIGN and SMS Malware classes, likely due to missing graph-based structure modeling. The GCN model misclassified SMS Malware and Adware, demonstrating its weakness in capturing sequence information—an issue DC-GL solves by combining LSTM. CapsNet-BiLSTM achieved relatively good performance overall, but its complex structure appears less effective in handling boundary cases, especially in distinguishing Scareware from Ransomware. While CT-GCN showed performance close to DC-GL, it still struggles to clearly separate Scareware and Adware, potentially due to limited sensitivity to fine-grained traffic features.

The HMM model performed the weakest among all evaluated methods. It showed high misclassification rates across multiple classes, particularly for Ransomware and SMS Malware. This is expected, as HMM’s probabilistic assumptions are less suited for high-dimensional and nonlinear encrypted traffic data.

DyGCN-LSTM improved upon traditional GCN and CNN-LSTM by dynamically updating the graph structure, but still showed some confusion between similar traffic categories due to its conventional convolution operations.

Transformer-CNN combined the strengths of Transformer and CNN, achieving good overall performance. However, it still exhibits moderate confusion between Benign traffic and SMS malware, indicating that the combination of residual connections and convolution has not fully resolved the challenge of distinguishing similar temporal patterns.

TransECA-Net performed stably across most categories, but still struggles to clearly differentiate between SMS malware and Benign traffic, suggesting room for improvement in capturing fine-grained temporal features.

In summary, the DC-GL model achieved better overall performance in multi-class detection by combining GCN for spatial modeling and LSTM for temporal learning, enhanced with detachable convolution and attention. This improves feature extraction, reduces confusion among similar traffic types, and boosts adaptability in complex scenarios.

#### Model convergence comparison

**Fig. 11:**
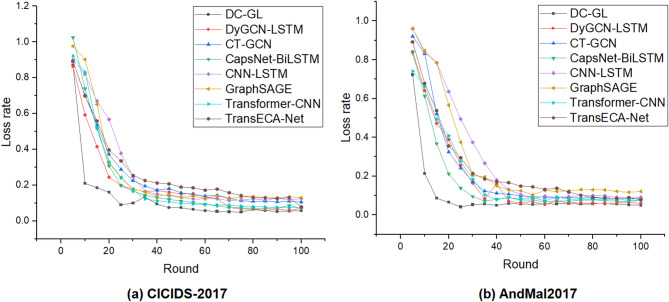
Fitted velocity comparison plots.

The DC-GL model was compared with several mainstream models, including GraphSAGE, CNN-LSTM, CapsNet-BiLSTM, CT-GCN, HMM, DyGCN-LSTM, Transformer-CNN, and TransECA-Net, on the CICIDS-2017 and AndMal2017 datasets in terms of training and inference time. The comparison results were presented in the Table 7, and the fitting speed comparison is illustrated in Fig. 11.

**Table 7:** Time efficiency result table.

Model	Time/epoch (s)	CICIDS-2017	AndMal2017
DC-GL	Training process	503.3	66.0
	Detecting process	208	2.3
	Overall	711.3	93.3
GraphSAGE	Training process	458.7	60.2
	Detecting process	118.1	15.5
	Overall	576.8	75.7
CNN-LSTM	Training process	523.5	68.7
	Detecting process	147.8	19.4
	Overall	671.3	88.1
CapsNet-BiLSTM	Training process	1,212.6	113.6
	Detecting process	363.8	34.1
	Overall	1,576.4	147.7
CT-GCN	Training process	641.6	84.2
	Detecting process	200.4	26.3
	Overall	842.0	110.5
HMM	Training process	339.6	22.9
	Detecting process	109.3	6.4
	Overall	448.9	29.3
DyGCN-LSTM	Training process	608.1	79.8
	Detecting process	188.3	24.7
	Overall	796.4	104.5
Transformer-CNN	Training process	1,027.5	96.3
	Detecting process	310.5	29.1
	Overall	1,338.0	125.4
TransECA-Net	Training process	1,079.7	101.2
	Detecting process	351.0	32.9
	Overall	1,430.7	134.1

In terms of computational efficiency, the DC-GL model demonstrate competitive training and inference speed. Although there is a performance gap when compared to lightweight traditional models such as HMM, DC-GL significantly outperforms other hybrid deep learning architectures—including Transformer-CNN, TransECA-Net, and CapsNet-BiLSTM—in terms of detection efficiency. On the CICIDS-2017 dataset, DC-GL required 503.3 s per training epoch, which is substantially lower than that of TransECA-Net (1079.7 s/epoch) and Transformer-CNN (1027.5 s/epoch). On the smaller AndMal2017 dataset, the model also maintained low computational overhead, indicating its efficiency across different data scales. All experiments were conducted using an NVIDIA GeForce RTX 4060 GPU. While the model performs well in GPU environments, inference latency increases significantly in CPU-only settings, highlighting the need for further optimization in resource-constrained deployments.

Regarding convergence speed, DC-GL achieved stable performance within the first 20 training rounds, showing a faster convergence rate than most baseline models. This can be attributed to its detachable graph convolution design, which effectively captures essential features early in the training process. In contrast, Models such as CNN-LSTM exhibited greater fluctuations in loss curves, and others like GraphSAGE converge more slowly.

It is worth noting that, despite DC-GL’s promising balance between performance and efficiency, certain challenges remain in real-world deployment scenarios, particularly in real-time detection settings where inference latency is critical. While DC-GL is well-suited for GPU-based systems, its practicality on edge or embedded devices remains limited. Future work may explore model compression, lightweight adaptation, or hardware-aware optimization to further reduce latency and meet the demands of low-latency, high-throughput environments.

#### Comparison of differentiation between positive and negative samples

**Fig. 12:**
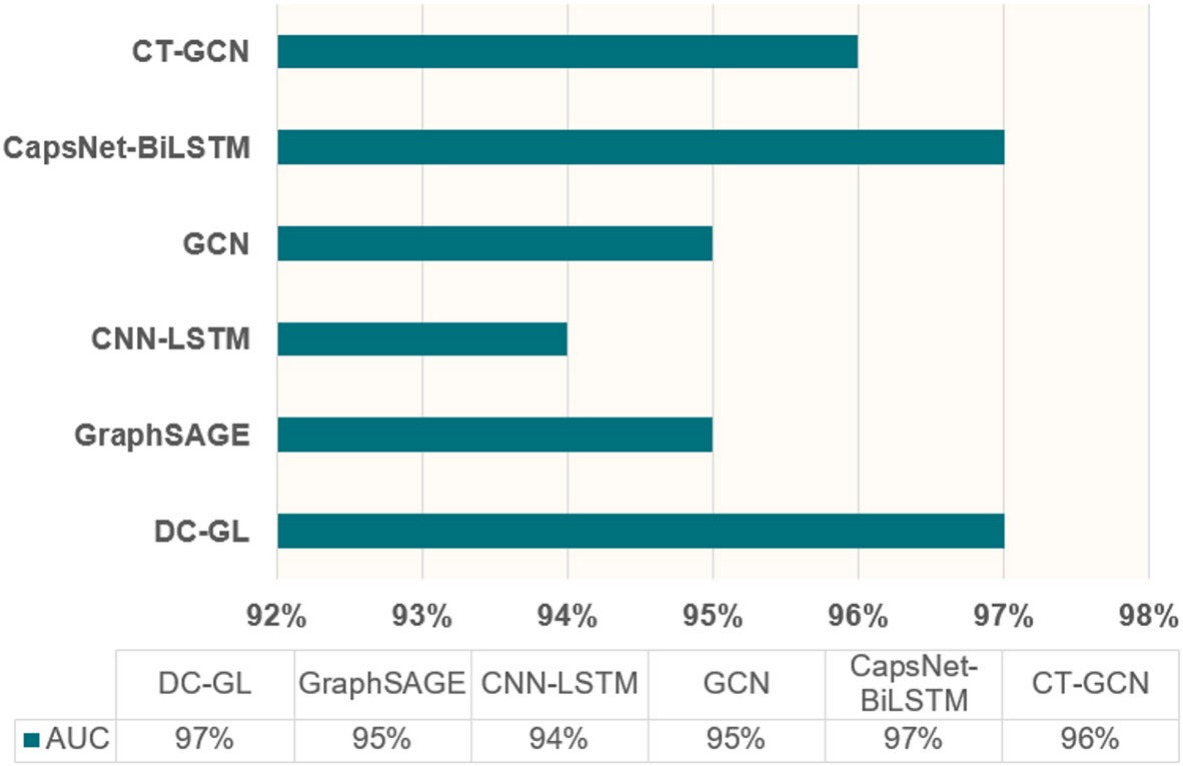
Comparison of AUC values.

To demonstrate the DC-GL model’s accuracy in distinguishing positive and negative samples, we compared it with the GCN model, CT-GCN model, CNN-LSTM model, CapsNet-BiLSTM model, and GraphSAGE model. The results are shown in Fig. 12.

Both DC-GL and CapsNet-BiLSTM achieved an AUC of 97%, outperforming the other models, as illustrated in Fig. 12. This indicates that the DC-GL model had a strong ability to distinguish between positive and negative samples under the given classification threshold. It achieved a high true positive rate while maintaining a low false positive rate. This is because detachable convolution extracts deep spatial features related to packet size, transmission interval, and other dimensions, and the LSTM layer memorizes and analyzes flow rate and connection frequency changes over time, integrating temporal features from previous and current time steps. Through the interaction of these features, the DC-GL model can more sensitively perceive subtle differences between normal and malicious traffic.

In contrast, the AUC was 96% for CT-GCN, 95% for GCN and GraphSAGE, and 94% for CNN-LSTM. These models also showed a good ability to distinguish positive and negative samples, but their performance is slightly inferior to the former two. As a result, they could lead to more misjudgments when facing more subtle or complex differences between positive and negative samples.

#### Analysis of model robustness

**Fig. 13:**
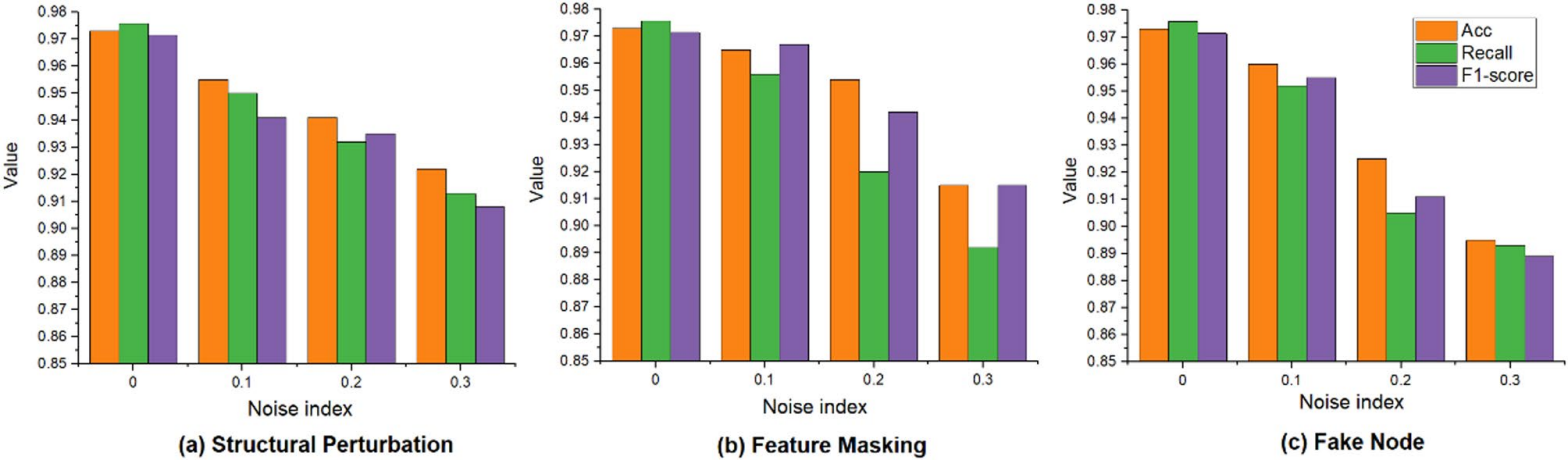
Comparison of results under different noises.

To comprehensively evaluate the robustness of the DC-GL model, three types of perturbations were introduced with gradually increasing intensity levels ranging from 0 to 30%: (1) structural perturbation, implemented by randomly flipping elements in the adjacency matrix to simulate topological noise; (2) feature masking, which randomly occludes portions of node features to mimic incomplete or missing input; and (3) fake node, where adversarial elements are added to the graph structure to simulate poisoning attacks. The performance under each perturbation scenario is illustrated in Fig. 13.

.

As shown in Fig. 13, the DC-GL model exhibited a relatively smooth degradation in performance as the perturbation intensity increased from 0 to 30% under all three types of interference: structural perturbation, feature masking, and fake nodes. Under structural noise, the model maintained around 92.5% accuracy at 30% edge flips, indicating strong robustness to topology changes. In the case of feature masking, even with 30% of the node features occluded, accuracy remained around 91%, showing good tolerance to incomplete input. Fake node injections, though the most adversarial, only reduced performance to about 89% accuracy. At full intensity, the model still performed effectively.

These results highlighted the robustness built into the model’s architecture. The detachable convolution decouples structure and feature learning, reducing sensitivity to edge perturbation; the LSTM module leverages temporal context to mitigate input corruption; and the attention mechanism adaptively filters noisy neighbors. Overall, DC-GL demonstrates strong resilience and is well-suited for deployment in complex or adversarial network environments.

## Conclusion

As malicious activities increasingly hide within encrypted traffic, the effectiveness of traditional detection methods continues to decline, and existing techniques often struggle to capture both structural and temporal features simultaneously. To address this challenge, this paper proposes a detection model that integrates a graph convolutional network based on detachable convolution with a long short-term memory network. The GCN captures structural dependencies, while the long short-term memory network models temporal dynamics. An attention mechanism is introduced to enhance feature representation. Compared to standard convolution, the use of detachable convolution significantly reduces computational complexity, accelerates training convergence, and improves overall efficiency. Experimental results demonstrate that the proposed model outperforms mainstream baseline methods on several key metrics. Compared with other complex deep fusion architectures, this model achieves a better balance between detection performance and computational cost, showing promise for practical application.

However, the graph construction process partly relies on protocol features from the Transport Layer Security, such as version, cipher suite, and server name indication. This assumption may limit the model’s generalization ability for emerging encryption protocols that do not expose similar handshake information. Future work will explore incorporating more protocol-independent statistical features or modeling unique structures of different protocols to extend the model’s applicability to various encrypted traffic scenarios.

Moreover, although the model performs well on public datasets, its scalability and robustness in real-world network environments require further validation. Future work will focus on collecting and labeling encrypted traffic from operational networks to build more representative datasets. The feasibility of deploying the model in real-time detection systems will also be explored—particularly with regard to controlling inference latency on low-resource devices—to ensure stable and low-latency performance under complex and dynamic network conditions.

## Data Availability

All data generated during and/or analyzed during the current study has been provided within the manuscript.
